# Plant community richness and foliar fungicides impact soil *Streptomyces* inhibition, resistance, and resource use phenotypes

**DOI:** 10.3389/fmicb.2024.1452534

**Published:** 2024-10-07

**Authors:** Matthew Michalska-Smith, Daniel C. Schlatter, Nuttapon Pombubpa, Sarah C. Castle, A. Stuart Grandy, Elizabeth T. Borer, Eric W. Seabloom, Linda L. Kinkel

**Affiliations:** ^1^Department of Plant Pathology, University of Minnesota, St. Paul, MN, United States; ^2^Department of Ecology, Evolution and Behavior, University of Minnesota, St. Paul, MN, United States; ^3^United States Department of Agriculture-Agricultural Research Service (USDA-ARS) Plant Science Research Unit, St. Paul, MN, United States; ^4^Department of Microbiology, Faculty of Science, Chulalongkorn University, Bangkok, Thailand; ^5^Department of Microbiology and Plant Pathology, University of California, Riverside, Riverside, CA, United States; ^6^Center for Biogeochemistry and Microbial Ecology (Soil BioME), University of New Hampshire, Durham, NC, United States; ^7^Department of Natural Resources and the Environment, University of New Hampshire, Durham, NC, United States

**Keywords:** soil nutrients, microbe-microbe interactions, phenotypes, co-evolution, phylogeny, plant diversity, soil ecology

## Abstract

Plants serve as critical links between above- and below-ground microbial communitites, both influencing and being influenced by microbes in these two realms. Below-ground microbial communities are expected to respond to soil resource environments, which are mediated by the roots of plants that can, in turn, be influenced by the above-ground community of foliar endophytes. For instance, diverse plant communities deposit more, and more diverse, nutrients into the soil, and this deposition is often increased when foliar pathogens are removed. Differences in soil resources can alter soil microbial composition and phenotypes, including inhibitory capacity, resource use, and antibiotic resistance. In this work, we consider plots differing in plant richness and application of foliar fungicide, evaluating consequences on soil resource levels and root-associated *Streptomyces* phenotypes. Soil carbon, nitrogen, phosphorus, potassium, and organic matter were greater in samples from polyculture than monoculture, yet this increase was surprisingly offset when foliar fungal communities were disrupted. We find that *Streptomyces* phenotypes varied more between richness plots—with the *Streptomyces* from polyculture showing lower inhibitory capacity, altered resource-use profiles, and greater antibiotic resistance—than between subplots with/without foliar fungicide. Where foliar fungicide affected phenotypes, it did so differently in polyculture than in monoculture, for instance decreasing niche width and overlap in monoculture while increasing them in polyculture. No differences in phenotype were correlated with soil nutrient levels, suggesting the need for further research looking more closely at soil resource diversity and particular compounds that were found to differ between treatments.

## 1 Introduction

While these two worlds can influence one another through myriad direct and indirect pathways, plants are critical links between above- and below-ground microbial ecosystems (Masters et al., 1993; Bardgett et al., 1998; Van der Putten et al., 2001; Bezemer and van Dam, 2005). In this work, we focus on the role of plants in facilitating biota in above-ground compartments to indirectly impact soil communities, by modifying plant productivity, growth, and morphology (Foley et al., 2021; Bagchi et al., 2017; Casas et al., 2011; Bardgett et al., 1998).

The phyllosphere represents a vast and functionally diverse microbial habitat, with complex effects on plant health and productivity. Plant-beneficial phyllosphere inhabitants can provide defense from pathogens or herbivores (Liu et al., 2020; Ritpitakphong et al., 2016), induce resistance (Bezemer and van Dam, 2005), produce plant hormones (Abadi et al., 2020; Lu et al., 2018), fix nitrogen (Madhaiyan et al., 2015), or confer resistance to abiotic stressors (Qu et al., 2020; Xu et al., 2022). However, foliar pathogens, especially fungi, often have strong negative effects on plant health and productivity and result in major crop losses worldwide (Teng et al., 1984).

Despite multiple studies focusing on herbivory by relatively large animals (insects, vertebrates), there are few data on linkages between foliar and soil microbial communities. Yet, impacts of foliar microbes on host plants are likewise expected to have consequences for below-ground communities. For example, fungicide treatments have been shown to increase above- and below-ground plant biomass (Seabloom et al., 2017). Luo et al. (2022) found that infection of *Panax notoginseng* with the foliar pathogen *Alternaria panax* enhanced root exudation of organic acids, sugars, and amino acids, as well as modified rhizosphere soil microbial community composition. Rudrappa et al. (2008) demonstrated that foliar infection of *Arabidopsis thaliana* by *Pseudomonas syringae* increased root exudation of D-malic acid and promoted root colonization by beneficial *Bacillus subtilis*. Over multiple growing seasons, changes in plant phenotypes or productivity because of plant-microbe interactions in the phyllosphere may modify soil resource concentrations (e.g., carbon, nitrogen, phosphorous, and potassium) in ways that can also impact soil microbial communities.

In contrast, plant host community richness has been repeatedly shown to exert broad influence on soil microbial communities through alteration of the above- and below-ground nutrient environments. Specifically, diverse plant communities are often substantially more productive (Tilman et al., 2001) and often experience altered pathogen effects (Mitchell et al., 2002; Seabloom et al., 2017) than monoculture. Moreover, plant richness has been hypothesized to be a crucial driver of soil bacterial community composition and function through the provision of greater abundances and diversities of plant-derived resources (Schlatter et al., 2015; Meier and Bowman, 2008; Bakker et al., 2013b; Ng et al., 2014). For example, greater abundances of carbon compounds support higher soil microbial densities, alter microbial community structure and diversity, and impact soil enzyme activities (Griffiths et al., 1998; Hernández and Hobbie, 2010). Further, greater diversity of carbon compounds in soil is hypothesized to increase the number of ecological niches available to soil microbes and has been found to increase microbial diversity and shift microbial carbon use capacities (Orwin et al., 2006; Essarioui et al., 2016; Kinkel et al., 2011).

In addition to ecological effects on soil microbes, resource environments likewise influence the co-evolutionary dynamics of microbial populations, including the structuring of inter-microbial interactions (Craig MacLean et al., 2005; Lawrence et al., 2012). For instance, Kinkel et al. (2011) have theorized that differing levels of disturbance and soil resource diversity can drive selection pressures for alternative evolutionary trajectories—favoring antagonism or niche differentiation (Pekkonen et al., 2013; Fiegna et al., 2015). Specifically, low-diversity resource environments, where microbes must compete for resource pools, are predicted to favor high frequencies of antagonistic microbes (e.g., antibiotic producers) and may generate a co-evolutionary arms-race (Bakker et al., 2013b; Kinkel et al., 2014, 2011). In contrast, a greater diversity of resources is hypothesized to promote adaptive radiation and niche differentiation among microbial taxa, as a means to minimize resource competition (Craig MacLean et al., 2005; Kinkel et al., 2014). Finally, greater resource abundance, by supporting higher microbial densities and encounter rates, may strengthen selection along either pathway, speeding the rate of co-evolution (Lopez Pascua et al., 2014).

*Streptomyces* are filamentous, gram-positive bacteria that are found ubiquitously in soils and are often closely associated with plant roots in the rhizosphere or endosphere (Viaene et al., 2016). As producers of antibiotic compounds, *Streptomyces* are important in clinical medicine and in suppressing soil-borne plant pathogens in natural and agricultural systems (Viaene et al., 2016; Barka et al., 2016; Schlatter and Kinkel, 2014; Jauri et al., 2016; Liu et al., 2019). In addition to their antibiotic-producing capacities, *Streptomyces* are a substantial natural reservoir of antibiotic resistance genes (D'Costa et al., 2007) and are highly diverse in their abilities to utilize resources for growth (Schlatter et al., 2013). Together, *Streptomyces* antibiotic inhibition, resistance, and resource use traits are all hypothesized to be critical for their interactions with other microbes, and in their capacities to respond to variation in resource inputs and impact plant health (Kinkel et al., 2014; Schlatter et al., 2009, 2013).

Variation in microbial interaction traits in soil are likely to reflect the abundance and diversity of soil resources, with consequences for microbial community diversity and function (Schlatter et al., 2009). Yet, despite the importance of plant-derived resources as a mechanistic link between plants and soil microbial communities, our understanding of the specific impacts of the abundance and diversity of plant-derived resources on traits of soil microbes in field settings remains limited. Here, we test how chronic disruption of foliar fungi (by fungicide applications) affects soil resources and *Streptomyces* interaction phenotypes (inhibition, resistance, and nutrient-use). We conduct this test in two communities that differ in plant richness (a monoculture and a polyculture with sixteen species). In agreement with previous research, we hypothesize that disruption of the foliar fungal community will increase below-ground carbon accumulation (Kohli et al., 2021; Zaret et al., 2022; Yang et al., 2009; Cong et al., 2014; Seabloom et al., 2017; but see Luo et al., 2022), and that these effects will be greater (in both absolute and relative terms) in diverse vs. monoculture plant communities (Seabloom et al., 2017). We expect these differences in soil nutrient levels to effect differences in *Streptomyces* community composition, as well as antibiotic inhibitory/resistance and resource use phenotypes, especially through an increase in inhibitory phenotypes in monoculture compared to more diverse plant communities (Bakker et al., 2013b). In accordance with the co-evolutionary theory put forward by Kinkel et al. (2011), we expect the disruption of the foliar fungal communities to favor escalation of antagonism, while an increase in plant (and therefore soil carbon) diversity will favor character displacement.

## 2 Methods

### 2.1 Experimental design and soil sampling

Soils for this study were collected in November 2013 from a long-term grassland biodiversity experiment at the University of Minnesota Cedar Creek Ecosystem Science Reserve (CCESR), part of the U.S Long Term Ecological Research (LTER) Network (45.4°N, 93.2°W). Soils at this site are sandy and nutrient poor (Grigal et al., 1974; Fay et al., 2015), and all topsoil was removed prior to the establishment of the biodiversity experiment (Tilman et al., 1997). The entire experimental field is burned each spring to remove any litter remaining from the previous year.

The two 9 × 9 m biodiversity plots from which our data were collected were established in 1994 (19 years prior to sampling); one was maintained as an *Andropogon gerardii* monoculture and the other was maintained as a 16 species native perennial polyculture containing *Andropogon gerardii*. In 2008, a foliar fungicide treatment [2 L m-2 Quilt (Syngenta Crop Protection, Inc.), a combination of Azoxystrobin (7.5 %) and Propiconazole (12.5 %)] applied biweekly) was nested into the plant diversity experimental plots to create 1.5 × 2 m fungicide treatment and control subplots within the 1 and 16 species whole plots. Care was taken in the application of the foliar fungicide to minimize contamination of the soil surface. In addition, Seabloom et al. (2017) found no direct effects of fungicide on plant biomass when grown in sterile soil. A more detailed description can be found in Borer et al. (2015), Seabloom et al. (2017). The current study sampled within the paired untreated control and fungicide treated subplots at each of the two levels of plant diversity.

Critically, due to the timing of fungicide application and sample collection, we do not interpret application of fungicide to mean foliar fungi were eliminated from these plants. While prior literature has highlighted the reduction in pathogenic fungi following fungicide application in these experimental plots (with consequent beneficial effects on host plants; Borer et al., 2015; Seabloom et al., 2017; Kohli et al., 2021), there has been less investigation into the consequences for foliar fungal communities as a whole. In other experimental systems, fungicide application was not found to reduce overall fungal diversity, richness, and colonization rate (Lane et al., 2023; Chen et al., 2020). In the absence of definitive evidence of the impact of our fungicidal treatment on the foliar community abundance and composition, in this work we consider this treatment as a temporary disruption of the foliar fungal community, with possible consequences for plant health and resource allocation.

Triplicate (1 cm diameter, 10 cm depth) soil cores were collected from within 10 cm of the central axis of 6 individual *Andropogon gerardii* plants from each subplot. Soil cores from the same individual plant were bulked in the field (*n* = 6 plants × 2 diversity treatments × 2 fungicide treatments = 24 composite samples), transported in a cooler to the lab, sieved (using a 2 mm mesh), and stored at -20 °C until processing. All processing, isolation, and phenotype characterization experiments took place in 2013–14.

### 2.2 *Streptomyces* densities and inhibitor densities

*Streptomyces* densities (total and proportion demonstrating inhibitory phenotypes) were determined as described previously (Bakker et al., 2013b). Briefly, soil samples were dried under two layers of sterile cheesecloth in a fume hood overnight. Dry soil samples were finely ground and 5 g of each soil was added to 25 mL of sterile deionized (DI) water and placed on an orbital shaker at 175 rpm at 4 °C for 60 min. Soil suspensions were serially diluted in sterile DI water and 100 μL of each dilution was spread onto 1 % water agar. After plates were allowed to dry, 5 mL of 1 % starch casein agar (SCA; Küster and Williams, 1964) was pipetted to cover the entire plate. Prior to overlaying, SCA was allowed to cool to prevent the medium from killing microorganisms. SCA is a semi-selective medium for *Streptomyces* and allows filamentous microbes to grow through the medium while suppressing non-filamentous bacteria (Oskay et al., 2009). Plates were incubated for 3 d at 28 °C and *Streptomyces* densities were evaluated by counting the number of colonies exhibiting characteristic *Streptomyces* morphology. After determining densities, a modified Herr's assay (Wiggins and Kinkel, 2005a,b) was used to assess inhibitory *Streptomyces* densities. Briefly, plates were overlaid with 10 mL of 1 % SCA, dried until solid, and then overlaid with 150 μL of spore suspension of an indicator strain (*Streptomyces* strains LK1324.2 or DL87). After 3 d of growth at 28 °C, *Streptomyces* colonies inhibiting the indicator overlay were counted and inhibition zone sizes were measured twice from the edge of the colony to the edge of clear overlay inhibition at right angles to one another. Overlay DL87 is a *Streptomyces scabies* isolate, which causes common scab disease of potato (Liu et al., 1996), and LK1324.2 is a non-pathogenic isolate previously obtained from CCESR soil (Davelos et al., 2004). For each composite soil sample, densities (CFU/g) were averaged over three replicate plates for each overlay isolate (*n* = 6 plates per soil sample).

### 2.3 *Streptomyces* isolation

After processing, soils from two individual plants/treatment (eight of the original 24 total bulked samples; two from each subplot) were randomly selected for *Streptomyces* isolation. Soils were serially diluted in sterile DI and 10 μL was spread on SCA. After 5 d of growth, colonies exhibiting characteristic *Streptomyces* morphology were selected by dividing each plate into a grid and randomly picking grid cells from which to select colonies. Five colonies were collected from three separate plates for each soil sample. *Streptomyces* colonies were picked with a sterile toothpick and streaked onto oatmeal agar (OA; Kharel et al., 2010). After 5 d of growth, single colonies were swabbed with a cotton applicator and spread as a lawn on OA. After ≈7 d growth, spores were collected by gently swabbing *Streptomyces* lawns with 4 mL of a 20 % glycerol solution. Spore stocks were stored at -20 °C until further use. Eighty *Streptomyces* isolates (*n* = 10 isolates per plant) were randomly selected for further characterization.

### 2.4 *Streptomyces* resource use characterization

For each *Streptomyces* isolate, resource use phenotypes on 95 distinct carbon sources were determined using Biolog SF-P2 plates (Biolog, Inc. Hayward, CA) as described previously (Schlatter et al., 2013). Briefly, fresh spore suspensions of *Streptomyces* isolates were adjusted to an OD_590_ of 0.22 and 100 μL of a spore suspension was inoculated into each well of a Biolog SF-P2 plate. After 3 d of growth at 28 °C, the absorbance in each well at 590 nm (AU590) was measured using a BioTek Synergy H1 plate reader (BioTek Instruments, Inc. Winooski, VT, USA). The absorbance in the water control well was subtracted from all other wells prior to subsequent analyses. A carbon source was considered to be used by an isolate if the AU590 was >0.01 above the water control well. Niche width and growth efficiency were determined for each isolate, where the niche width of an isolate is the number of used resources and resource use efficiency is the mean absorbance value for used resources. Niche overlap, a measure of shared resource use among isolates, was calculated for each pair of isolates *a* and *b* as the average pairwise niche overlap: ${\bar{\omega }}_{a\to b}=\frac{1}{n}\overset{n}{\underset{i}{\sum }}\frac{min\left(od_{a}^{\left(i\right)},od_{b}^{\left(i\right)}\right)}{od_{a}^{\left(i\right)}}$, where $od_{x}^{\left(i\right)}$ is the absorbance (i.e., growth) of isolate *x* on carbon source *i*. Notably, this metric is asymmetric, such that, in general, ${\bar{\omega }}_{a\to b}\neq {\bar{\omega }}_{b\to a}$.

### 2.5 16S rRNA gene sequencing

Partial 16S rRNA gene sequences were obtained for *n* = 76 isolates using previously described protocols (Davelos et al., 2004). Briefly, genomic DNA was extracted from cultures of each isolate grown in Yeast-Dextrose broth for 3 d (28 °C, 175 rpm) using the Wizard Genomic DNA Purification kit (Promega Corp., Madison, WI). PCR reactions consisted of 12.5 μL HotStart Master Mix (Qiagen), 0.75 μL of pA (10 pM), 0.75 μL of pH (10 pM), 1.0 μL of *Streptomyces* DNA (25 ng), and 10.0 μL of PCR grade H_2_O. Thermocycling conditions consisted of an initial denaturation of 95 °C for 4 min, followed by 30 cycles of 95 °C for 30 s, 50 °C for 30 s, 72 °C for 90 s, and a final extension at 72 °C for 7 min. PCR products were checked for the expected product on a 1 % agarose gel, purified with the Qiaquick PCR cleanup kit (Qiagen, Valencia, CA), and sequenced using the forward primer (pA) at ACGT, Inc (Wheeling, IL). Sequences were edited manually, classified using the RDP Classifier (Wang et al., 2007), and aligned using MUSCLE (v3.8.1551) (Edgar, 2004). Gaps were removed using trimAl (v1.4.rev22) (Capella-Gutiérrez et al., 2009) and a maximum likelihood phylogenetic tree was constructed using RAxML (v8.2.12) (Stamatakis, 2014) using a GTR+γ+I substitution model, *Embleya hyalina* strain NBRC 13850 as an outgroup, and 1,000 bootstraps to assess confidence. Phylogenetic diversity of isolates from each treatment was quantified with Faith's Phylogenetic Diversity using the pd function in the picante (v. 1.8.2) R package (Faith, 1992).

### 2.6 Antibiotic resistance

Resistance profiles against nine clinical antibiotics [kanamycin (30 μg), streptomycin (10 μg), erythromycin (15 μg), vancomycin (30 μg), amoxicillin/clavulanic acid (30 μg), novobiocin (5 μg), chloramphenicol (30 μg), rifampin (5 μg), and tetracycline (30 μg) (BD BBL Sensi-Disc; Becton, Dickinson and Company, Sparks, MD)] were determined for each isolate using a disk-diffusion assay as detailed in Otto-Hanson et al. (2013). Five of these antibiotics are produced by *Streptomyces*; and their modes of action include inhibition of protein, RNA, DNA gyrase, and cell wall synthesis.

In brief, 100 μL of each isolate was spread on plates containing 15 mL of SCA. Four different antibiotic discs were then applied to each plate and plates were incubated at 27 °C for 3 d. Inhibition zones were calculated by averaging two right-angle radial measurements. Each isolate-antibiotic combination was replicated twice.

### 2.7 Soil nutrient characteristics

Total soil carbon (C; %) and nitrogen (N; %) were determined for each composite soil sample (≈20 g/sample) at the University of Nebraska-Lincoln Ecosystem Analysis Laboratory using a Costech ECS 4010 elemental analyzer (Costech Analytical Technologies, Inc.). Soil (≈20 g/sample) from each composite sample was analyzed for soil organic matter (%), Bray-1 extracted phosphorus (P; ppm), NH_4_OAc extracted potassium (K; ppm), and pH at the University of Minnesota Research Analytical Laboratory (https://ral.cfans.umn.edu) using standard protocols.

### 2.8 Statistical analysis

Variance in phylogenetic (unweighted unifrac) distance was partitioned across treatments (plant diversity and fungicide application) with a permutational multivariate analysis of variance using the adonis2 function in the vegan (v. 2.6-4) R package (Oksanen et al., 2022). Differences in *Streptomyces* populations (total densities, inhibitory densities, and proportions), resource use (niche width, mean efficiency, niche overlap), and soil characteristics with plant richness and fungicides were assessed with a nested ANOVA, where the fungicide factor was nested within plant richness. All statistical analyses were performed in R (v. 4.3.1) (R Core Team, 2023) unless otherwise indicated.

## 3 Results

In this work, we examine differences in phenotypes across 80 *Streptomyces* isolates collected from plots that vary in plant richness and disruption of the foliar fungal community via fungicide application. Specifically, the fungicide treatment is nested within the richness plots, leading to a hierarchical design. We consider phylogenetic relatedness, inhibitory resource-use, and antibiotic phenotypes, as well as their relationship to *in situ* soil nutrient levels. In these results, we generally present statistical differences using *post-hoc* test results, focusing on the nested interaction between fungicide and plant richness; however, in some cases, we instead focus on differences between richness treatments that do not appear clearly when fungicide is additionally taken into account. Importantly, *Streptomyces* community composition is expected to vary seasonally, and in response to the pulse perturbation of foliar fungicide. Thus, our results represent a snapshot of time within these wider dynamics. Also note that, though we have replicated plants within each plot, our experimental design contains only one plot for each level of plant richness. As such, care must be taken to not overinterpret these results and to consider them in the context of the wider literature on plant richness and fungicide effects on soil *Streptomyces* communities.

### 3.1 *Streptomyces* community composition

**Figure 1:**
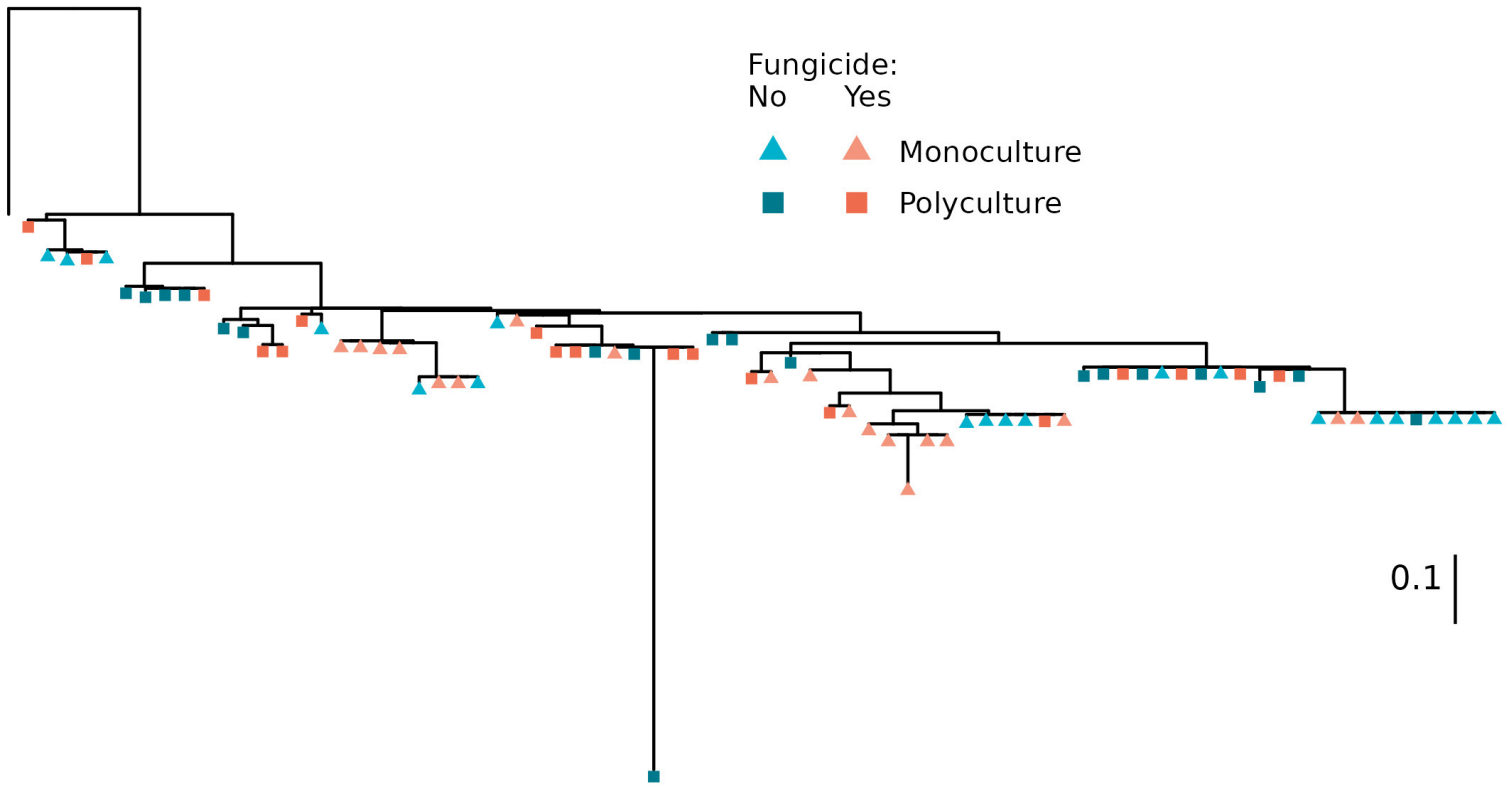
Phylogenetic relationships between isolates. Isolates are colored according to the plant richness of the sample plot and shaped according to foliar-community disruption via application of a foliar fungicide. Tree was generated with RAxML (v8.2.12) (Stamatakis, 2014) using a GTR+γ+I substitution model, *Embleya hyalina* strain NBRC 13850 as an outgroup (leftmost, unlabeled tip), and 1,000 bootstraps to assess confidence. The scale bar indicates evolutionary distance (substitution/site). Branch support values and isolate identity can be found in Supplementary Figure S1.

The composition of *Streptomyces* communities differed among treatments (Figure 1; *p* = 0.048). When comparing plant richness or fungicide treatments, communities were significantly phylogenetically clustered according to plant richness (*p* = 0.020), but not disruption of foliar communities within monoculture or polyculture (*p* = 0.458). If the one outlying isolate is removed, monoculture communities were found to be more phylogenetically diverse than communities from polyculture (*p* = 0.003), but the effect of fungicide more nuanced, with untreated polyculture communities being less diverse than monoculture communities, and those from fungicide treated polyculture falling in between.

### 3.2 Soil nutrient characteristics

**Figure 2:**
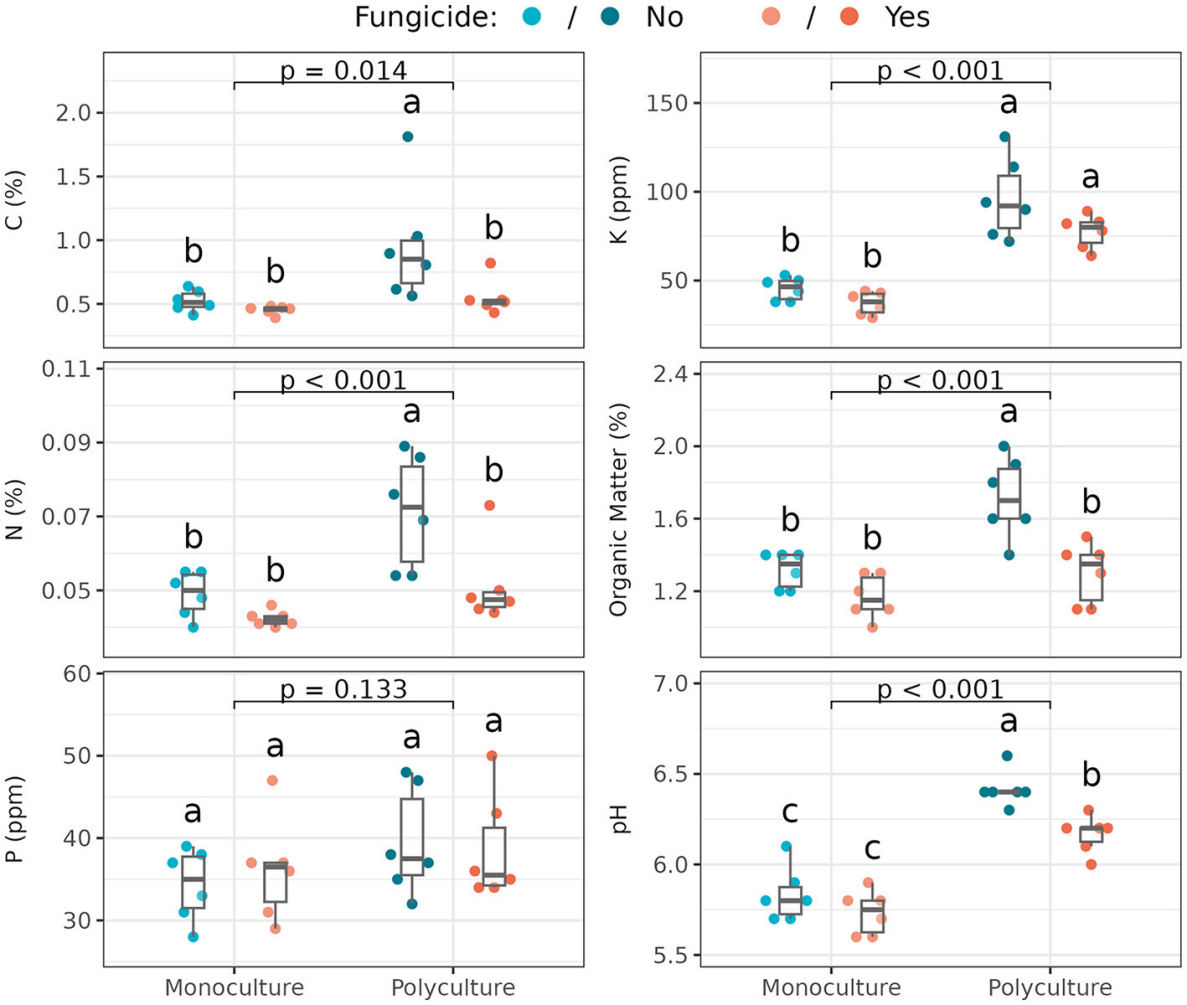
Soil nutrient characteristics among plant richness and fungicide treatments (*n* = 6 samples per treatment, i.e,. subplot). Box plots indicate 25th, 50th, and 75th percentiles of the data, with whiskers extending to the largest (smallest) value no further than 1.5 times the interquartile range beyond the 75th (25th) percentile. The reported *p*-value is for the plant richness effect, while different letters above boxplots indicate statistically significant differences between all four subplots using Tukey's HSD *post-hoc* test (*p* < 0.05). Means and standard deviations for each measure, as well as full ANOVA results are reported in Supplementary Tables S1, S2.

Soil resources [total carbon (C; %), total nitrogen (N; %), and organic matter (%)], as well as soil pH in the rooting zone of *Andropogon gerardii* varied significantly with foliar community disruption, but only in polyculture (Figure 2; Supplementary Table S1). Surprisingly, in polyculture, the untreated subplot tended to have greater C, N, and organic matter than the treated subplot, and than both treated and untreated monoculture. Soil in the monoculture plot was more acidic than polyculture soil. Moreover, in polyculture, the fungicide-treated subplot had lower pH (that is, more similar to monoculture) than untreated ones. Potassium (K; ppm) was higher in polyculture than monoculture, but with no differences between foliar fungicide treatments, and phosphorus (P; ppm) was unchanged across all treatments. In summary, resource abundance was found to vary significantly across treatments (Supplementary Table S2): polyculture had higher resource abundances than monoculture, and foliar fungal disruption was more influential in polyculture, where untreated polyculture samples had the highest resource abundances and treated polyculture samples were usually indistinguishable from monoculture samples.

### 3.3 *Streptomyces* densities and inhibitory activities and their relationships with soil nutrient characteristics

**Figure 3:**
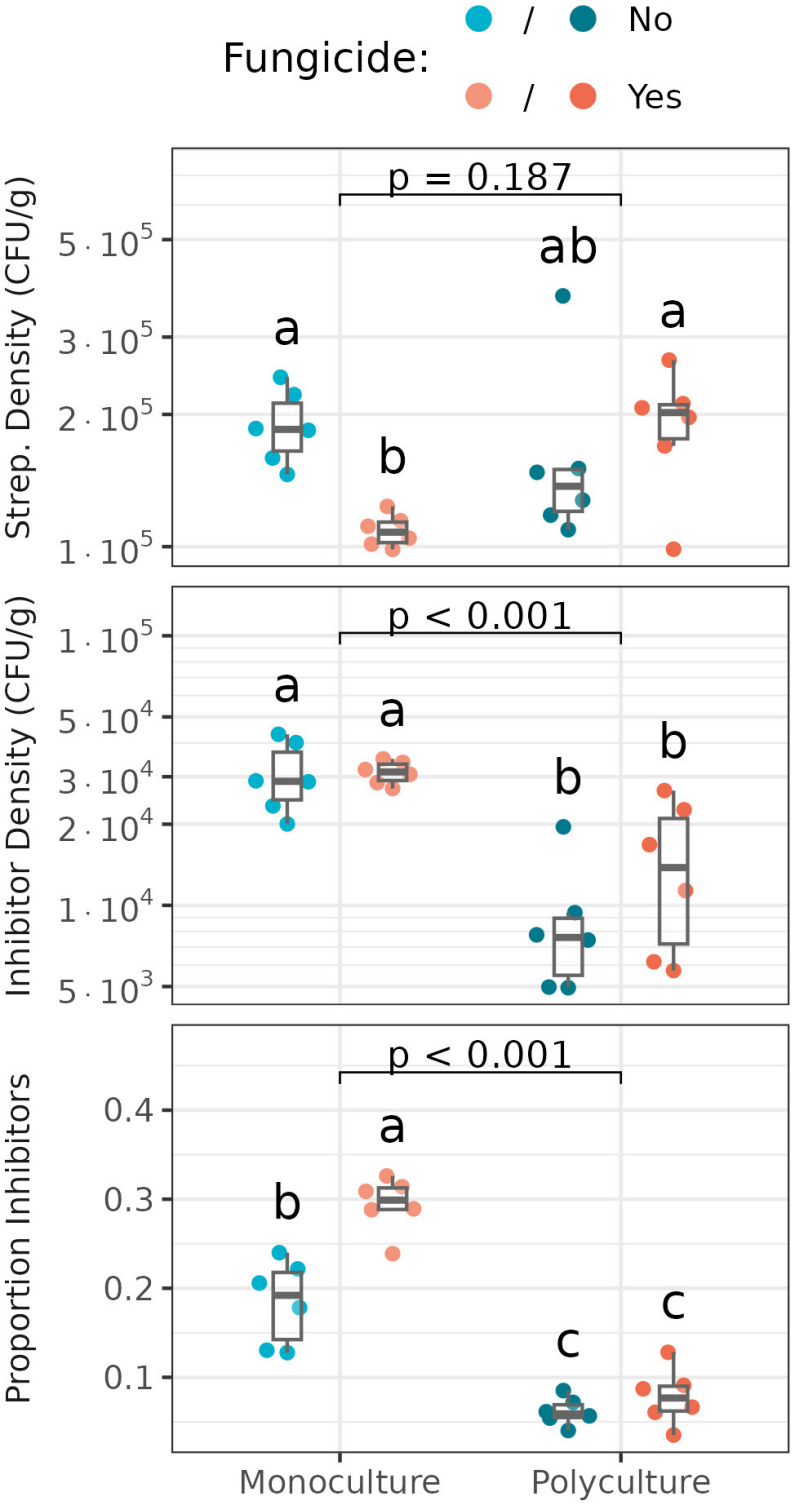
Densities of *Streptomyces* **(Top)**, inhibitory *Streptomyces* **(Middle)**, and proportions of *Streptomyces* that are inhibitory **(Bottom)** in untreated (blue) and fungicide-treated (orange) monoculture and polyculture. Box plots indicate 25th, 50th and 75th percentiles of the data, with whiskers extending to the largest (smallest) value no further than 1.5 times the interquartile range beyond the 75th (25th) percentile. The reported *p*-value is for the plant richness effect, while different letters above boxplots indicate statistically significant differences between all four subplots using Tukey's HSD *post-hoc* test (*p* < 0.05). Means and standard deviations for each measure, as well as full ANOVA results are reported in Supplementary Tables S7, S8.

Total *Streptomyces* densities and densities of inhibitory *Streptomyces* differed significantly among plant richness and fungicide treatments (Figure 3). *Streptomyces* densities were lowest in monoculture whose foliar fungal community was disrupted and higher in treated polyculture and untreated monoculture (Figure 3, Top). Considering only *Streptomyces* exhibiting inhibitory phenotypes, the monoculture plot supported significantly higher densities of inhibitors than did the polyculture plot, though there was no significant effect of foliar fungicide treatment on inhibitor densities in either plant richness treatment (Figure 3, Middle). Proportions of *Streptomyces* exhibiting inhibitory phenotypes varied significantly with both plant richness and disruption of foliar fungal communities (Figure 3, Bottom). Inhibitory *Streptomyces* comprised ≈ 10–20% more of the total *Streptomyces* community in monoculture vs. polyculture. Disruption of foliar fungal communities further increased the relative frequency of inhibitory *Streptomyces* in monoculture, but not in polyculture. Together, these data suggest that antibiotic-producing *Streptomyces* are selectively enriched in monoculture vs. polyculture settings, and that disruption of foliar community structure increases the selection for antibiotic inhibition, but only in monoculture (Essarioui et al., 2017). In contrast with densities and proportions of inhibitory *Streptomyces*, mean inhibition zone sizes, or the intensity of *Streptomyces* inhibitory phenotypes, did not vary significantly among treatments (data not shown).

Correlations between *Streptomyces*/inhibitor densities and soil nutrient characteristics differed between polyculture and monoculture plots. In monoculture, soil C, N, K, and organic matter were all positively correlated with *Streptomyces* densities (Pearson's *R*^2^ ≥0.36, *p* < 0.041; Supplementary Table S3). In contrast, the proportion of *Streptomyces* that exhibited inhibitory phenotypes were significantly negatively correlated with N and organic matter in monoculture (*R*^2^ ≥0.42, *p* < 0.022; Supplementary Table S3). In polyculture, however, there was only one significant correlation between *Streptomyces* density metrics and soil nutrients: a negative relationship between the density of inhibitors and soil P (*R*^2^ = 0.53, *p* = 0.017; Supplementary Table S4). This suggests that *Streptomyces* densities and inhibitory phenotypes in polyculture are less responsive to soil resource levels than those in monoculture.

Interestingly, the proportions of inhibitory *Streptomyces* were negatively correlated with overall *Streptomyces* densities in monoculture (*R*^2^ = 0.81, *p* < 0.001), but not polyculture (*R*^2^ = 0.05, *p* = 0.490), while absolute densities of inhibitors showed the opposite trend, significantly increasing with *Streptomyces* density in polyculture (*R*^2^ = 0.64, *p* = 0.002), but not in monoculture (*R*^2^ = 0.02, *p* = 0.691). Taken together, these suggest that as *Streptomyces* abundance increases in monoculture, non-inhibitory *Streptomyces* increase faster than their inhibitory counterparts, resulting in non-inhibitory *Streptomyces* representing a larger proportion of the total *Streptomyces* community than they did at lower abundances. However, in polyculture, the relative frequency of inhibitory *Streptomyces* remains largely constant, with both inhibitory and non-inhibitory *Streptomyces* increasing in tandem.

### 3.4 Nutrient use and niche overlap among *Streptomyces* isolates

**Figure 4:**
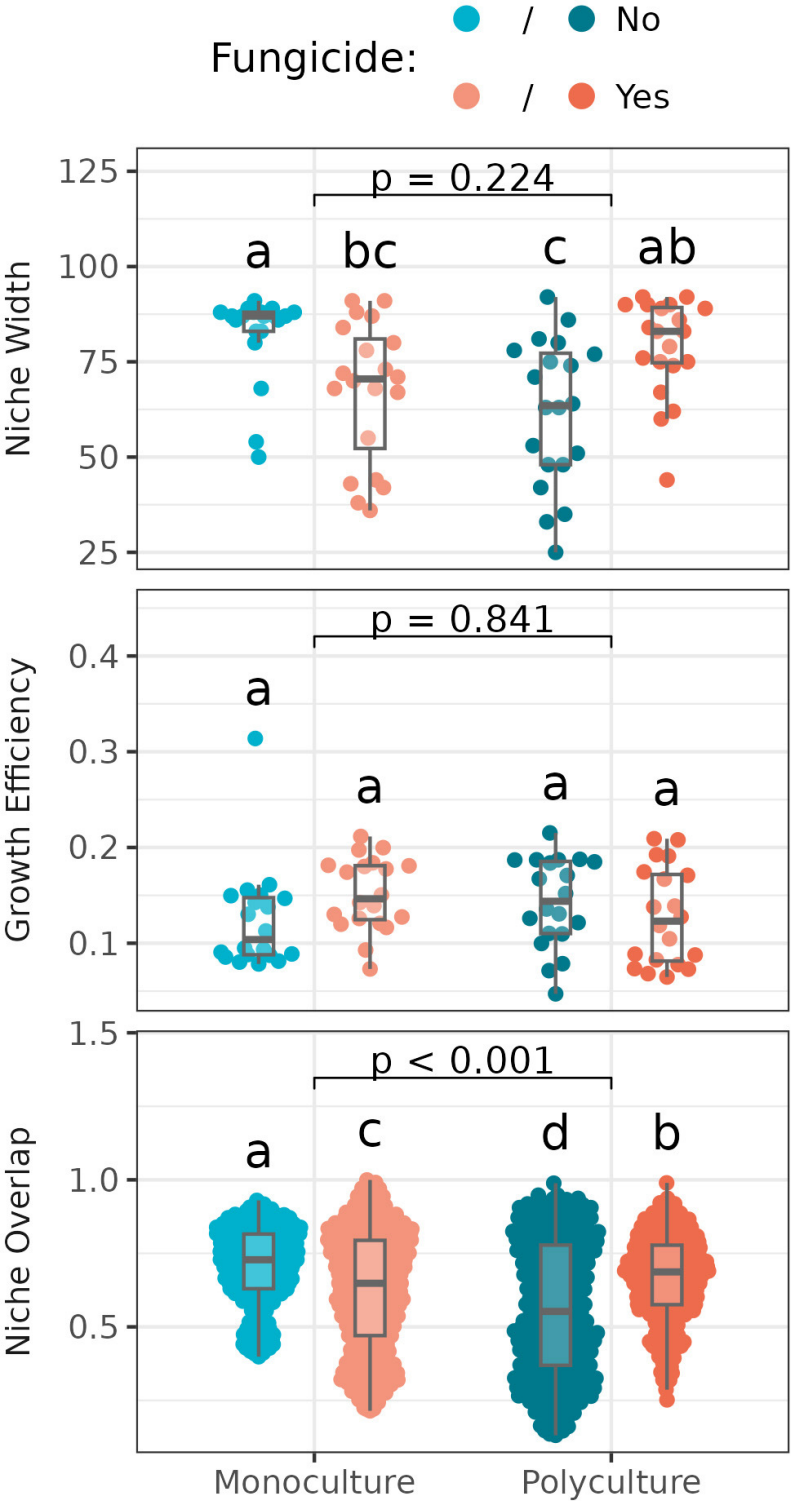
Niche width (number of carbon sources utilized; **Top**), growth efficiency (average optical density across utilized carbon sources; **Middle**), and niche overlap (shared use of carbon sources between paired isolates; **Bottom**) among *Streptomyces* from different plant richness and fungicide treatments. Box plots indicate 25th, 50th, and 75th percentiles of the data, with whiskers extending to the largest (smallest) value no further than 1.5 times the interquartile range beyond the 75th (25th) percentile. The reported *p*-value is for the plant richness effect, while different letters above boxplots indicate statistically significant differences between all four subplots using Tukey's HSD *post-hoc* test (*p* < 0.05). Means and standard deviations for each measure, as well as full ANOVA results are reported in Supplementary Tables S9, S10.

Niche width, or the number of resources that individual *Streptomyces* could grow on, varied significantly with disruption of the foliar fungal community, but not with plant richness. However, the effect of this disruption on *Streptomyces* niche widths differed between monoculture vs. polyculture (Figure 4). Specifically, in the monoculture subplot where foliar fungicides were applied, *Streptomyces* had reduced niche widths compared to the subplot with undisturbed foliar fungal communities, while in polyculture, niche widths were increased where foliar fungicide was applied.

Growth efficiency, or the average growth of individual *Streptomyces* isolates across used resources, did not vary significantly with plant richness or fungicide treatment (Figure 4). Niche overlap, or the degree to which isolates sampled from the same plant grow on the same resources, varied significantly across both foliar fungicide and plant richness treatments (Figure 4), following a similar pattern as noted for niche width (with which niche overlap is strongly correlated; *R*^2^ = 0.79, *p* < 0.001).

### 3.5 Antibiotic resistance

**Figure 5:**
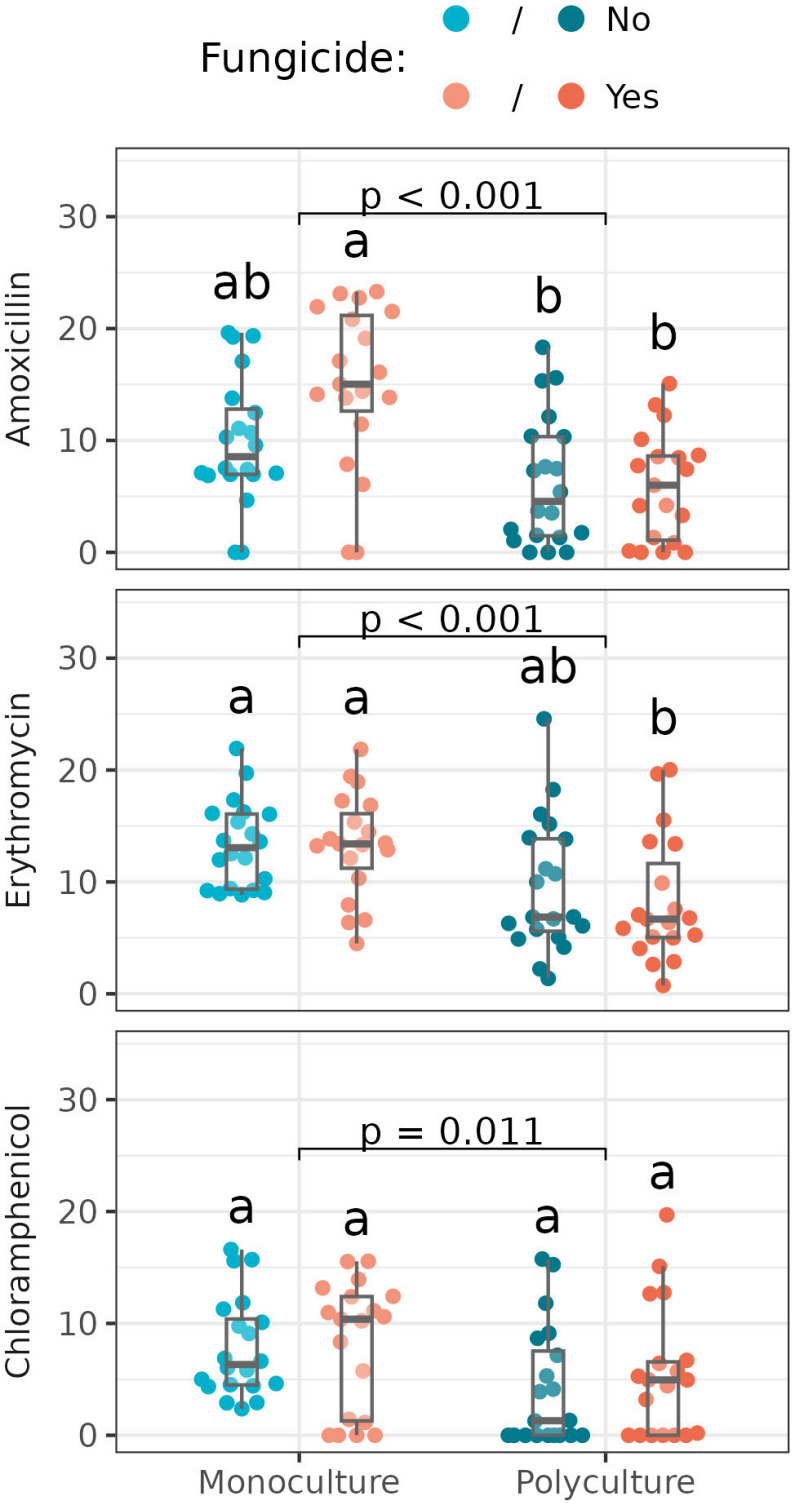
Resistance zone sizes of *Streptomyces* isolates from different plant richness and fungicide treatments (*n* = 18–20 isolates/treatment). Box plots indicate 25th, 50th, and 75th percentiles of the data, with whiskers extending to the largest (smallest) value no further than 1.5 times the interquartile range beyond the 75th (25th) percentile. The reported *p*-value is for the plant richness effect, while different letters above boxplots indicate statistically significant differences between all four subplots using Tukey's HSD *post-hoc* test (*p* < 0.05). Antibiotics without significant differences across treatments (and all means and standard deviations) are shown in Supplementary Table S5. An analysis of variance reports a significant difference between *Streptomyces* from monoculture vs. polyculture for all three antibiotics listed here, with more resistance (i.e., smaller resistance zone sizes) in *Streptomyces* from polyculture (Supplementary Table S6).

For six of the nine antibiotics tested, we found no significant difference in inhibition zone size across treatments (Supplementary Tables S5, S6). For amoxicillin, erythromycin, and chloramphenicol, however, we found *Streptomyces* from monoculture were significantly more susceptible (i.e., had larger inhibition zones in response to antibiotic disks) than those from polyculture (N.b. that the difference in chloramphenicol was significant in an analysis of variance despite no significant pairwise differences found in the *post-hoc* analysis; Figure 5; Supplementary Table S6).

We found additional effects of foliar fungicide application on isolate susceptibility to amoxicillin: isolates from subplots in which the foliar fungal communities were disrupted were more susceptible to amoxicillin, compounding the difference seen between plant richnesses (Figure 5). Yet, in all three cases, more variance was explained by the plot-level richness treatment than disruption of the foliar fungal community (Supplementary Figure S2).

### 3.6 Differentially used nutrients among *Streptomyces*

**Figure 6:**
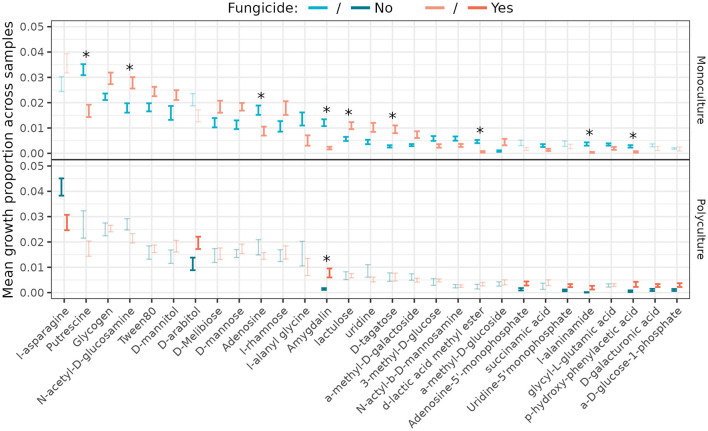
Proportion of total resource use by *Streptomyces* isolates from different plant richness (monoculture—top, polyculture—bottom) and fungicide (yes—orange, no—blue) treatments (*n* = 20 isolates/treatment). Error bars represent standard error around the mean. Significant (*p* < 0.05) pairwise differences are darker/thicker than non-significant comparisons, and those comparisons that remained significant following correction for multiple comparisons are marked with an asterisk. Only carbon sources for which the difference across fungicide treatments was significant for at least one level of plant richness were included. See Supplementary Figure S3 for all 95 carbon sources.

*Streptomyces* growth rates differed between fungicide treatments for twenty-three carbon sources in monoculture and nine in polyculture (Figure 6; Supplementary Figure S3); however only seven of the significant relationships in monoculture (and none in polyculture) remained significant following correction for multiple hypothesis testing by controlling the false discovery rate (Benjamini and Hochberg, 1995; Benjamini and Yekutieli, 2001). Five of those seven showed a reduction in *Streptomyces* growth on the carbon source when foliar fungal communities were disrupted. Likewise, Fifteen comparisons were significant across plant richness treatment (Supplementary Figure S4), with none significant after correction. Applying a nested analysis of variance structure as used elsewhere in this work, we found nine carbon sources for which richness was significant, and sixteen for which disruption of the foliar fungal community (nested within richness) was significant (Supplementary Figures S5, S6).

## 4 Discussion

The role of plant hosts and microbial species interactions in mediating selection for microbial phenotypes in soil remains poorly understood. Plant species richness is associated with both greater above-ground plant productivity and increased soil nutrient abundance and diversity (Tilman et al., 2001; Ng et al., 2014; Steinauer et al., 2016; El Moujahid et al., 2017; Yang et al., 2019; Furey and Tilman, 2021), in turn producing microbial communities with greater niche differentiation and increased inhibitory phenotypes in monoculture compared to polyculture plots (Orwin et al., 2006; Essarioui et al., 2016; Bakker et al., 2013b). These data suggest a key role for species interactions, modulated by plant species richness, in determining a microbial community's co-evolutionary trajectory (Kinkel et al., 2011).

Here we leverage a long-term experiment in which plant productivity in both monoculture and polyculture was enhanced through regular disruption of foliar fungal communities to test the hypothesis that enhanced plant productivity heightens selection for antagonistic vs. niche-differentiated *Streptomyces* populations. We hypothesized that plant productivity-driven increases in nutrient fluxes to soil microbes drive niche differentiation as an alternative to antagonistic phenotypes, which are posited to provide a competitive advantage in low-nutrient soils. Consistent with theoretical expectations, we found greater niche differentiation (Kinkel et al., 2011) and reduced inhibitory phenotypes (Bakker et al., 2013b) among *Streptomyces* from higher plant richness. However, despite enhanced above-ground productivity associated with foliar fungal community disruption, effects on *Streptomyces* populations were nuanced. Disruption of foliar fungal communities in monoculture increased inhibitory phenotypes and reduced niche width and niche overlap, while in polyculture, this treatment had no effect on inhibition and increased niche widths and niche overlap. Moreover, contrary to our expectations, none of the observed differences in *Streptomyces* inhibitory, resource use, and resistance phenotypes could be correlated to any soil resource measures.

Critically, our experimental design contains only one plot for each level of plant richness. While replicate samples were taken within each subplot, this design constrains our inference as to the importance of plant richness as a driver of microbial phenotypes, rather than, for example, a plot effect. Nevertheless, there is substantial evidence that plant richness is a key factor differentiating *Streptomyces* phenotypes in these plots (Steinauer et al., 2016; El Moujahid et al., 2017; Schlatter et al., 2015; Bakker et al., 2013b; Essarioui et al., 2017, 2016; Bakker et al., 2013a, 2010), and that it has consequences for soil nutrient diversity (Steinauer et al., 2016; El Moujahid et al., 2017) and abundance (Yang et al., 2019; Furey and Tilman, 2021). Moreover, we see consistent differences in *Streptomyces* phenotypes across fungicide treatments within the polyculture plot, even though the treated subplot has resource levels largely indistinguishable from either of the monoculture subplots. Thus, while we cannot exclude the possibility that plot effects contribute to the observed resource and phenotypic differences among *Streptomyces*, we focus here on changes in the context of differing plant richness.

### 4.1 Soil nutrients vary with plant richness and foliar fungicide

As expected, we found higher resource levels in soil from the *Andropogon gerardii* rhizosphere in polyculture compared to monoculture (Anacker et al., 2021; Lange et al., 2015; Furey and Tilman, 2021), in particular in measures of carbon, nitrogen, organic matter, and potassium. Work in these same experimental plots has shown that polycultures produce more diverse carbon compounds in the soil (Supplementary Figure S7), in agreement with prior literature (Steinauer et al., 2016; El Moujahid et al., 2017). Yet, we saw either no appreciable change in (in monoculture) or a decrease in total nutrient abundance (and pH) following fungicide application (in polyculture), in contrast to prior literature (Seabloom et al., 2017). Importantly, the soil carbon metrics used in this work may fail to capture highly-labile carbon substrates that are rapidly metabolized by soil microbes, or the diversity of carbon substrates, either one of which can differ with plant species richness. A more detailed look at the soil chemistry, perhaps focused on compounds for which we saw differences in *Streptomyces* consumption across treatments, in the future could confirm the inferences put forth here.

For carbon, nitrogen, and organic matter, however, the disruption of the foliar fungal communities had the opposite effect to expectation in polyculture: offsetting the relative increase from monoculture and producing nutrient levels similar to those observed in the monoculture. Considering pH, soil from polyculture was significantly less acidic than monoculture, with foliar community disruption reducing this difference (making soil more acidic), but not as acidic as monoculture. This could suggest an overall positive effect of foliar fungi on soil nutrients in polyculture [as has, for instance, been noted for endophytic and rhizospheric fungi (Van Der Heijden et al., 2016; Baron and Rigobelo, 2022; Chetia et al., 2019) and even, in some cases, pathogens (Luo et al., 2022)], one which is mitigated by the application of foliar fungicide. We did not find an effect of foliar fungal community disruption on any measure of soil nutrients in monoculture. However, in light of extensive previous literature demonstrating the effects of plant richness (Steinauer et al., 2016; Furey and Tilman, 2021; Anacker et al., 2021; Lange et al., 2015; El Moujahid et al., 2017) and foliar fungicide application (Luo et al., 2022; Zaret et al., 2023) on soil resource levels, it is possible we do not see it here in part due to spatio-temporal heterogeneity.

### 4.2 Soil nutrients do not explain most differences in *Streptomyces* phenotypes

In line with theoretical expectations suggesting that microbes are less likely to invest in direct competition when resources are plentiful (Kinkel et al., 2011; Granato et al., 2019), we see lower levels of inhibition and niche overlap in polyculture (Bakker et al., 2013b). Interestingly, however, we also saw significant changes in soil microbial phenotypes that were independent of changes in soil resource abundance. For instance, while most measures of soil chemistry did not differ under monoculture between fungicide treatments, we nevertheless observed differences in *Streptomyces* densities, proportion of inhibitory *Streptomyces*, niche width, and niche overlap. For *Streptomyces* densities and the proportion of *Streptomyces* that were inhibitory, the disruption of foliar fungal communities in monoculture appeared to preferentially reduce the densities of non-inhibitory *Streptomyces*: leaving the total number of inhibitors unchanged, but substantially increasing their relative abundance. In contrast, the polyculture did not exhibit different proportions of inhibitory *Streptomyces*, but did show differences in nutrient abundance, with higher resource abundance (untreated subplot) corresponding to narrower niches, in contrast with prior work (Lin et al., 2021; Dundore-Arias et al., 2019). Importantly, our assessment of inhibitory capacity was limited to two indicator strains, and the addition of additional strains might alter our conclusions.

Despite no difference in *Streptomyces* abundance between fungicide treated/untreated subplots in monoculture, *Streptomyces* abundance was nevertheless positively correlated with greater soil nutrients, while the proportion of inhibitory *Streptomyces* decreased with greater soil resources (there were few significant correlations in polyculture). Consistent with previous work, these data suggest the significance of constitutive antibiotic production to *Streptomyces* fitness is lower in high resource environments (Bakker et al., 2013b); however, plant richness has been shown to increase both resource abundance and diversity (Steinauer et al., 2016; Yang et al., 2019; Furey and Tilman, 2021; El Moujahid et al., 2017), somewhat confounding our analyses.

### 4.3 *Streptomyces* phenotypes varied more across plant richness than foliar fungicide

Rather than foliar fungicide application or soil nutrient abundance, most variation in *Streptomyces* inhibition, nutrient-use, and resistance in our experiment was between the two plots differing in the richness of their host plant communities. In particular, we found that *Streptomyces* clustered phylogenetically according to source plot/plant richness and that the monoculture plot had both more a more diverse community (Schlatter et al., 2015) and consistently had higher rates of inhibition (Bakker et al., 2013b), higher levels of pairwise niche-overlap, and less resistance to specific antibiotics than did *Streptomyces* from polyculture (Bakker et al., 2010; Vetsigian et al., 2011).

This discordance between inhibitory and resistance phenotypes builds on current understanding about the relationship between diversity and microbial antagonistic phenotypes. One explanation could be the relative fitness costs associated with maintaining resistance vs. inhibitory capacity (Gerardin et al., 2016; Czárán et al., 2002). If the former is more costly in the face of the diversity of antibiotics one's neighbors could produce, it might be selectively lost in favor of strong offensive capabilities in a resource-rich environment (Vetsigian et al., 2011). Alternatively, taken together with changes in niche width/overlap in monoculture, this could indicate a community-level trade-off between maintaining broad niches and inhibitory (but not resistance) phenotypes (Schlatter and Kinkel, 2015). However, the absence of this relationship in *Streptomyces* from polyculture argues against this interpretation. Finally, at least some of these resistance phenotypes could be evolutionary spandrels, serving primarily some other purpose in *Streptomyces*, but having the convenient side effect of detoxification (González-Candelas et al., 2011; Fajardo et al., 2008). Further research could explore this more comprehensively by, for instance, seeing whether these results are consistent when resistance is evaluated with respect to antibiotics produced by sympatric *Streptomyces*, rather than standard clinical antibiotics.

If this dominating effect of plot is driven by plant richness, it would be both interesting and in agreement with results in the study of disease-suppressive soils and, in particular, monoculture decline (Papavizas and Lumsden, 1980), where sustained low-diversity plant communities dramatically impact microbial interaction phenotypes and the functioning of soil communities (Weller et al., 2002). Suppressive soils develop in agricultural systems after long-term monoculture where high densities and/or frequencies of antagonistic microbes accumulate and limit plant disease development (typically species of *Streptomyces, Pseudomonas, Bacillus*, or *Trichoderma*; Schlatter et al., 2017). In contrast, introducing plant richness (temporally) with crop rotation can disrupt the development of disease-suppressive soil communities (Schlatter et al., 2017). Similarly, in experimental prairie plant richness manipulations, the soils of long-term monocultures contain higher frequencies of pathogen-antagonists than more diverse plant communities. This reduced soil antagonist community with increasing plant richness may feedback to contribute to plant fitness in polyculture (Bakker et al., 2013b; Essarioui et al., 2016). Though the mechanisms of general pathogen suppression are poorly understood, both ecological and evolutionary dynamics are likely to be crucial to the generation of disease-suppressive microbial communities.

### 4.4 Conclusion

*Streptomyces* phenotypes of inhibition, antibiotic resistance, and nutrient-use differed across our treatments. We found that soil from polyculture tended to have higher resource abundances and diversities than monoculture, resulting in *Streptomyces* populations with lower proportions of inhibitors and less competition for resources (i.e., less niche overlap). Yet, many phenotypic differences appeared to be unrelated to our measures of soil resource abundance. Disrupting the foliar fungal community did not result in the expected increase in measured soil resources in monoculture, and led to reduced soil resources in polyculture. Nevertheless, we saw differences in soil *Streptomyces* inhibition and resource use phenotypes across fungicide treatments in monoculture, suggesting that disruption of the above-ground, foliar fungal community has ramifications for below-ground microbes beyond the resource pathways we focused on here. Moreover, the effect of foliar fungal disruption on *Streptomyces* resource use in monoculture (reduced niche width and overlap) was opposite for *Streptomyces* from polyculture (increased niche width and overlap). The relatively larger and more consistent phenotypic effects across plant richness as compared to fungicide treatment highlights the importance of plants in linking above- and below-ground ecosystems, as well as suggesting a potentially greater role for soil resource diversity over soil resource abundance in phenotypic selection.

## Data Availability

The data presented in the study are deposited in the Environmental Data Initiative (https://portal.edirepository.org/nis/mapbrowse?scope=knb-lter-cdr&identifier=736), doi: 10.6073/pasta/f921a2a9ad3eba4789c9ec7fe8bc1ab7.
