# Supplementary material for: Plant community richness and foliar fungicides impact soil Streptomyces inhibition, resistance, and resource use phenotypes
Source: Front Microbiol. 2024 Oct 7;15:1452534. doi: 10.3389/fmicb.2024.1452534 (PMC11491370; doi:10.3389/fmicb.2024.1452534)
Supplement: Supplementary file 1 [file Data_Sheet_1.PDF]

Supplementary Material

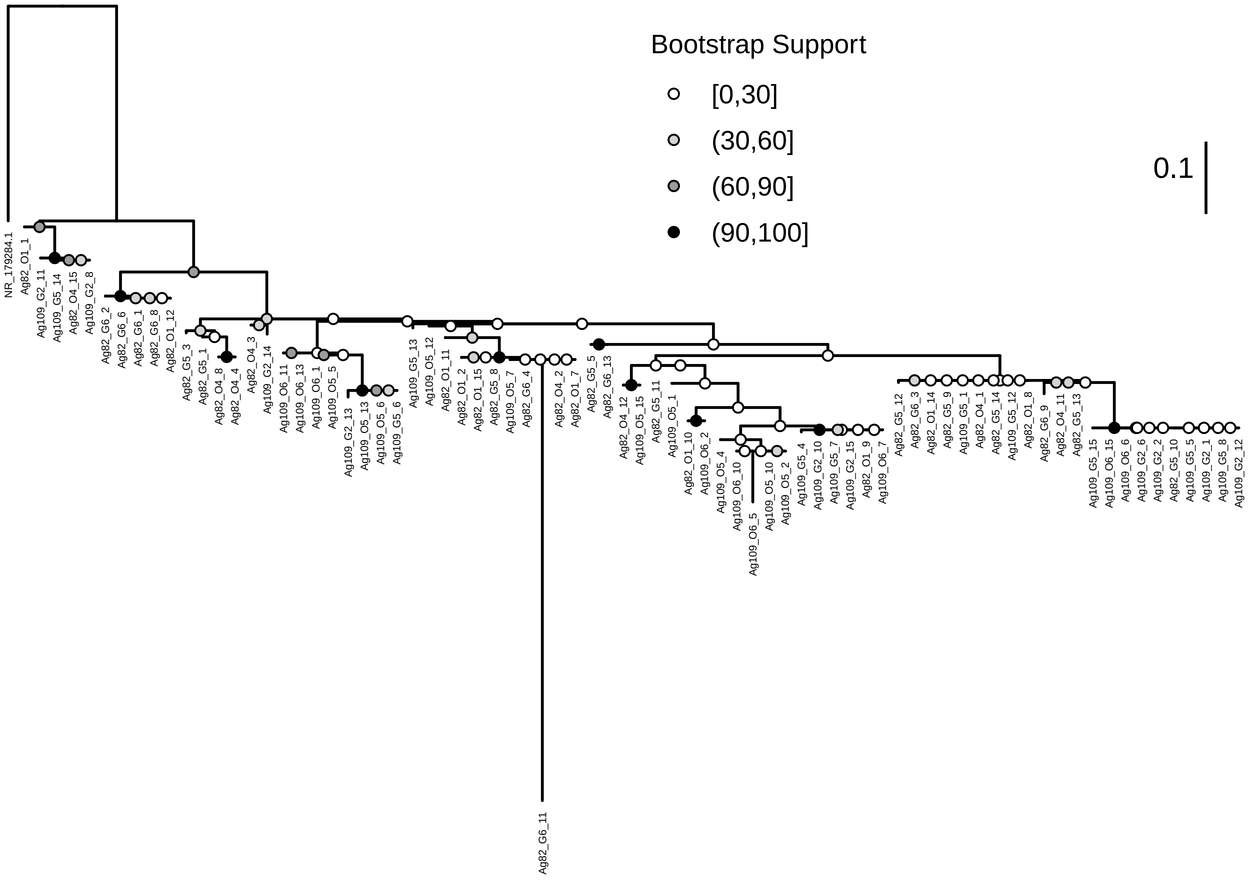

Figure S1. The tree here is identical in structure to that in Figure 1, with tips now coded in the format "Ag[DIVERSITY TREATMENT/PLOT]_[FUNGICIDE TREATMENT][PLANT WITHIN PLOT]_[ISOLATE NUMBER]". With 109 corresponding to monoculture and 82 polyculture, and G to untreated and O to fungicide treatment. Additionally, branch points are now shaded according to bootstrap support.

Table S1. Mean (± standard deviation) edaphic characteristics among plant richness and fungicide treatments (n = 6 samples per treatment). Differing letters signify significant ($p < 0.05$) differences according to a Tukey's Honestly Significant Difference post hoc test of an Analysis of Variance.

Plant Richness	Fungicide	C (%)	N (%)	P (ppm)	K (ppm)	Organic Matter (%)	pH
Monoculture	No	0.52 ± 0.08 b	0.05 ± 0.006 b	34.33 ± 4.4 a	45.33 ± 6.4 b	1.32 ± 0.1 b	5.83 ± 0.2 c
	Yes	0.45 ± 0.03 b	0.04 ± 0.002 b	36.17 ± 6.3 a	37.17 ± 6.4 b	1.17 ± 0.1 b	5.73 ± 0.1 c
Polyculture	No	0.95 ± 0.5 a	0.07 ± 0.01 a	39.50 ± 6.5 a	96.17 ± 22.6 a	1.72 ± 0.2 a	6.42 ± 0.1 a
	Yes	0.55 ± 0.1 b	0.05 ± 0.01 b	38.67 ± 6.5 a	77.50 ± 9.4 a	1.30 ± 0.2 b	6.17 ± 0.1 b

Table S2. Raw analysis of variance results for edaphic measures, considering the effect of plant richness and foliar fungicide (nested within the richness treatment).

	term	df	sumsq	meansq	statistic	*p*	
C (%)	plant richness	1	0.42	0.42	7.25	0.014	*
	plant richness : fungicide	2	0.50	0.25	4.26	0.029	*
	Residuals	20	1.17	0.06	NA	NA	
K (ppm)	plant richness	1	12467.04	12467.04	73.11	< 0.001	***
	plant richness : fungicide	2	1245.42	622.71	3.65	0.044	*
	Residuals	20	3410.50	170.53	NA	NA	
N (%)	plant richness	1	0.00	0.00	14.86	< 0.001	***
	plant richness : fungicide	2	0.00	0.00	6.90	0.005	**
	Residuals	20	0.00	0.00	NA	NA	
Organic Matter (%)	plant richness	1	0.43	0.43	16.73	< 0.001	***
	plant richness : fungicide	2	0.59	0.29	11.54	< 0.001	***
	Residuals	20	0.51	0.03	NA	NA	
P (ppm)	plant richness	1	88.17	88.17	2.46	0.133	
	plant richness : fungicide	2	12.17	6.08	0.17	0.845	
	Residuals	20	717.00	35.85	NA	NA	
pH	plant richness	1	1.55	1.55	107.54	< 0.001	***
	plant richness : fungicide	2	0.22	0.11	7.54	0.004	**
	Residuals	20	0.29	0.01	NA	NA	

Table S3. Pearson's correlation R^2-values and *p*-values among edaphic characteristics and *Streptomyces* densities in monoculture. Sign was preserved to indicate direction of relationship. Significant correlations are indicated by trailing *'s, with *** corresponding to a *p*-value < 0.001, ** to *p*-values < 0.01, and * to *p*-values < 0.05.

	C (%)	N (%)	P (ppm)	K (ppm)	Organic Matter (%)	pH
log(Streptomyces)	0.36 *	0.53 **	-0.03	0.42 *	0.49 *	0.08
log(Inhibitors)	-0.01	-0.03	-0.10	0.00	-0.04	0.05
Mean Kill Zone (mm)	-0.01	-0.01	-0.18	-0.12	-0.03	0.04
Proportion Inhibitors	-0.27	-0.42 *	0.00	-0.32	-0.46 *	-0.06

Table S4. Pearson's correlation R^2-values and *p*-values among edaphic characteristics and *Streptomyces* densities in polyculture. Sign was preserved to indicate direction of relationship. Significant correlations are indicated by trailing *'s, with *** corresponding to a *p*-value < 0.001, ** to *p*-values < 0.01, and * to *p*-values < 0.05.

	C (%)	N (%)	P (ppm)	K (ppm)	Organic Matter (%)	pH
log(Streptomyces)	0.12	0.09	-0.32	0.01	0.07	0.00
log(Inhibitors)	-0.03	-0.05	-0.53 *	-0.03	-0.02	0.02
Mean Kill Zone (mm)	0.07	0.08	-0.40	-0.10	0.02	-0.01
Proportion Inhibitors	-0.25	-0.29	-0.17	-0.08	-0.20	0.04

Table S5. Mean (± standard deviation) resistance zone sizes of *Streptomyces* isolates from different plant richness and fungicide treatments (n = 18 - 20 isolates/treatment). Different letters following means indicate significant differences among treatments using Tukey's post-hoc test ($p < 0.05$).

Plant Richness	fungicide	Amoxicillin	Chloramphenicol	Erythromycin	Kanamycin	Novobiocin	Rifampin	Streptomycin	Tetracycline	Vancomycin
Monoculture	Yes	14.86 ± 7.2 a	8.05 ± 5.8 a	13.27 ± 4.6 a	17.75 ± 5.7 a	8.35 ± 2.8 a	2.93 ± 4.5 a	15.29 ± 4.4 a	2.14 ± 2.0 a	12.31 ± 2.5 a
	No	9.89 ± 5.7 ab	7.82 ± 4.5 a	13.30 ± 3.8 a	16.07 ± 6.7 a	6.54 ± 4.1 a	1.64 ± 2.0 a	15.50 ± 3.8 a	1.76 ± 1.8 a	10.69 ± 3.0 a
Polyculture	No	6.24 ± 5.8 b	4.18 ± 5.3 a	9.50 ± 5.9 ab	16.17 ± 6.2 a	7.26 ± 3.0 a	2.00 ± 2.7 a	14.60 ± 4.1 a	1.62 ± 3.3 a	10.29 ± 4.3 a
	Yes	5.86 ± 4.8 b	5.37 ± 5.9 a	8.32 ± 5.6 b	17.84 ± 7.6 a	8.69 ± 3.8 a	3.19 ± 2.8 a	13.61 ± 4.1 a	3.34 ± 3.3 a	11.74 ± 2.8 a

Table S6. Raw analysis of variance results for *Streptomyces* resistance to nine antibiotics, considering the effect of plant richness and foliar fungicide (nested within the richness treatment).

Antibiotic	term	df	sumsq	meansq	statistic	p	
Amoxicillin	plant richness	1	762.81	762.81	21.60	< 0.001	***
	plant richness : fungicide	2	242.49	121.24	3.43	0.038	*
	Residuals	74	2613.74	35.32	NA	NA	
Chloramphenicol	plant richness	1	196.10	196.10	6.78	0.011	*
	plant richness : fungicide	2	14.24	7.12	0.25	0.782	
	Residuals	74	2139.20	28.91	NA	NA	
Erythromycin	plant richness	1	371.28	371.28	14.52	< 0.001	***
	plant richness : fungicide	2	13.70	6.85	0.27	0.766	
	Residuals	74	1892.59	25.58	NA	NA	
Kanamycin	plant richness	1	0.19	0.19	0.00	0.948	
	plant richness : fungicide	2	54.41	27.21	0.63	0.536	
	Residuals	74	3204.02	43.30	NA	NA	
Novobiocin	plant richness	1	5.59	5.59	0.47	0.495	
	plant richness : fungicide	2	51.97	25.98	2.19	0.119	
	Residuals	74	878.83	11.88	NA	NA	
Rifampin	plant richness	1	1.95	1.95	0.20	0.654	
	plant richness : fungicide	2	30.08	15.04	1.56	0.216	
	Residuals	74	712.03	9.62	NA	NA	
Streptomycin	plant richness	1	32.02	32.02	1.92	0.170	
	plant richness : fungicide	2	9.89	4.94	0.30	0.744	
	Residuals	74	1232.15	16.65	NA	NA	
Tetracycline	plant richness	1	5.09	5.09	0.71	0.401	
	plant richness : fungicide	2	30.40	15.20	2.13	0.126	
	Residuals	74	526.93	7.12	NA	NA	
Vancomycin	plant richness	1	4.59	4.59	0.44	0.509	
	plant richness : fungicide	2	46.24	23.12	2.22	0.116	
	Residuals	74	771.45	10.43	NA	NA	

Table S7. Mean (± standard deviation) resistance zone sizes of *Streptomyces* isolates from different plant richness and fungicide treatments (n = 18 - 20 isolates/treatment). Different letters following means indicate significant differences among treatments using Tukey's post-hoc test ($p < 0.05$).

Plant Richness	fungicide	Mean Kill Zone (mm)	Proportion Inhibitors	log(Inhibitors)	log(Streptomyces)
Monoculture	No	4.38 ± 0.9 a	0.18 ± 0.05 b	4.47 ± 0.1 a	5.27 ± 0.08 a
	Yes	3.90 ± 1.0 a	0.29 ± 0.03 a	4.49 ± 0.04 a	5.04 ± 0.04 b
Polyculture	Yes	4.15 ± 1.0 a	0.08 ± 0.03 c	4.10 ± 0.3 b	5.26 ± 0.1 a
	No	3.92 ± 1.2 a	0.07 ± 0.01 c	4.01 ± 0.2 b	5.25 ± 0.2 ab

Table S8. Raw analysis of variance results for *Streptomyces* community inhibition phenotypes, considering the effect of plant richness and foliar fungicide (nested within the richness treatment).

Edaphic measure	term	df	sumsq	meansq	statistic	p	
Mean Kill Zone (mm)	plant richness	1	0.04	0.04	0.04	0.845	
	plant richness : fungicide	2	0.80	0.40	0.40	0.677	
	Residuals	18	18.03	1.00	NA	NA	
Proportion Inhibitors	plant richness	1	0.17	0.17	155.82	< 0.001	***
	plant richness : fungicide	2	0.04	0.02	16.88	< 0.001	***
	Residuals	20	0.02	0.00	NA	NA	
log(Inhibitors)	plant richness	1	1.38	1.38	37.49	< 0.001	***
	plant richness : fungicide	2	0.12	0.06	1.62	0.222	
	Residuals	20	0.74	0.04	NA	NA	
log(Streptomyces)	plant richness	1	0.03	0.03	1.87	0.187	
	plant richness : fungicide	2	0.18	0.09	5.43	0.013	*
	Residuals	20	0.34	0.02	NA	NA	

Table S9. Mean (± standard deviation) nutrient use metrics of *Streptomyces* isolates from different plant richness and fungicide treatments (n = 20 isolates/treatment for niche width and growth efficiency; n =). Different letters following means indicate significant differences among treatments using Tukey's post-hoc test ($p < 0.05$).

Plant Richness	fungicide	Niche Width	Growth Efficiency	Niche Overlap
Monoculture	Yes	67.30 ± 18.3 bc	0.15 ± 0.04 a	0.63 ± 0.2 c
	No	82.30 ± 11.4 a	0.12 ± 0.05 a	0.71 ± 0.1 a
Polyculture	No	61.95 ± 19.2 c	0.14 ± 0.05 a	0.56 ± 0.2 d
	Yes	79.00 ± 12.8 ab	0.13 ± 0.05 a	0.67 ± 0.1 b

Table S10. Raw analysis of variance results for *Streptomyces* nutrient-use metrics, considering the effect of plant richness and foliar fungicide (nested within the richness treatment).

Nutrient-use metric	term	df	sumsq	meansq	statistic	p	
Growth Efficiency	plant richness	1	0.00	0.00	0.04	0.841	
	plant richness : fungicide	2	0.01	0.00	2.19	0.119	
	Residuals	76	0.17	0.00	NA	NA	
Niche Overlap	plant richness	1	0.90	0.90	27.75	< 0.001	***
	plant richness : fungicide	2	2.88	1.44	44.57	< 0.001	***
	Residuals	1368	44.15	0.03	NA	NA	
Niche Width	plant richness	1	374.11	374.11	1.51	0.224	
	plant richness : fungicide	2	5157.02	2578.51	10.37	< 0.001	***
	Residuals	76	18889.35	248.54	NA	NA	

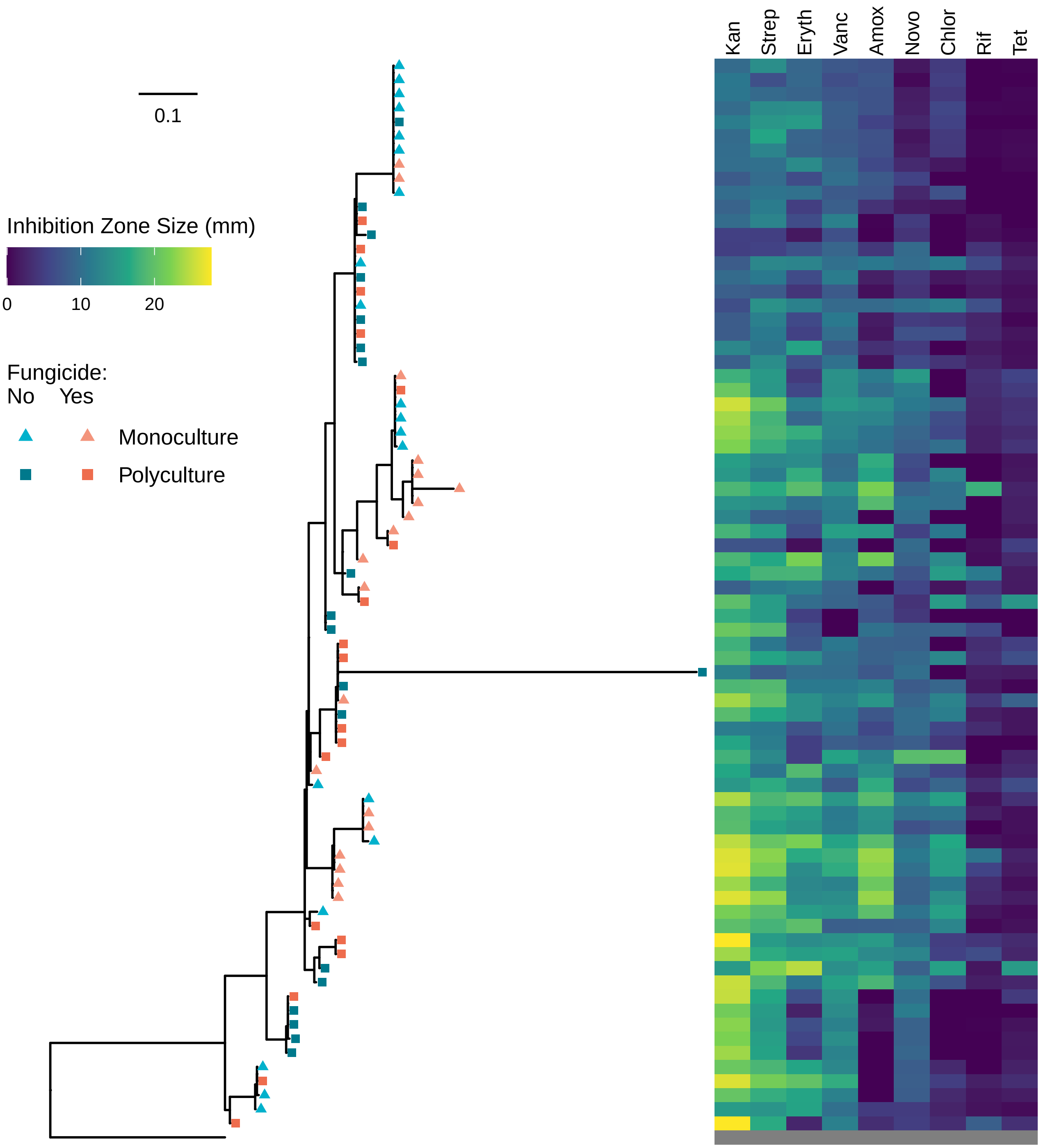

Figure S2. Antibiotic resistance profiles of each isolate, ordered according to phylogenetic relatedness. The tree here is identical to that in Figure 1, while each cell in the heatmap corresponds to the radius of that isolates inhibition zone size in millimeters to each of nine standard clinical antibiotics.

770 `Warning: Duplicated 'override.aes' is ignored.`

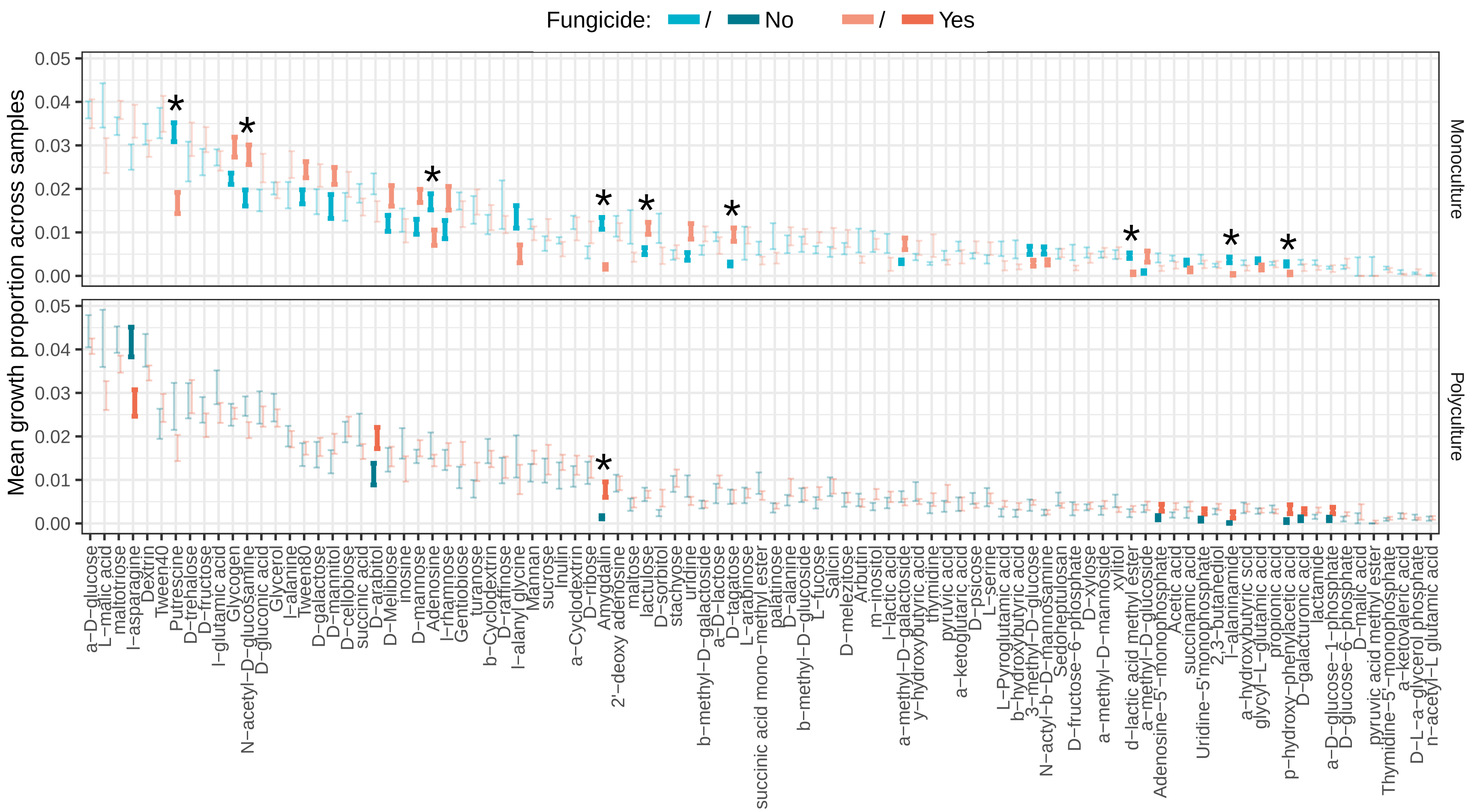

Figure S3. As Figure 6, but showing all 95 carbon sources. Proportion of total resource use by *Streptomyces* isolates from different plant richness and fungicide treatments (n = 20 isolates/treatment). Error bars represent standard error around the mean. Significant ($p < 0.05$) pairwise differences are darker/thicker than non-significant comparisons, and those comparisons that remained significant following correction for multiple comparisons are marked with an asterisk.

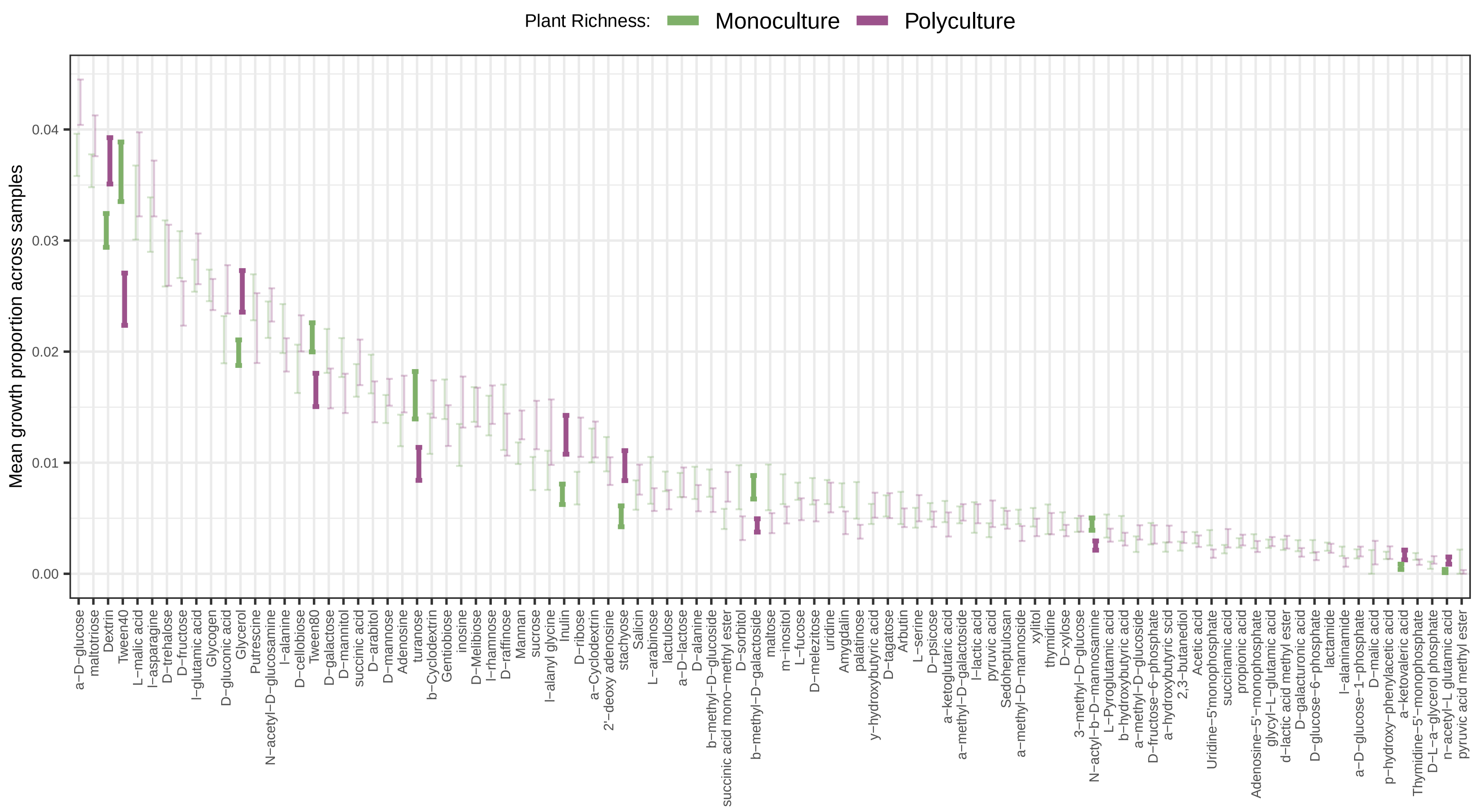

Figure S4. Proportion of total resource use by *Streptomyces* isolates from different plant richness treatments (n = 36 - 40 isolates/treatment). Error bars represent standard error around the mean. Significant ($p < 0.05$) pairwise differences are darker/thicker than non-significant comparisons, but none of these comparisons remained significant following correction for multiple comparisons.

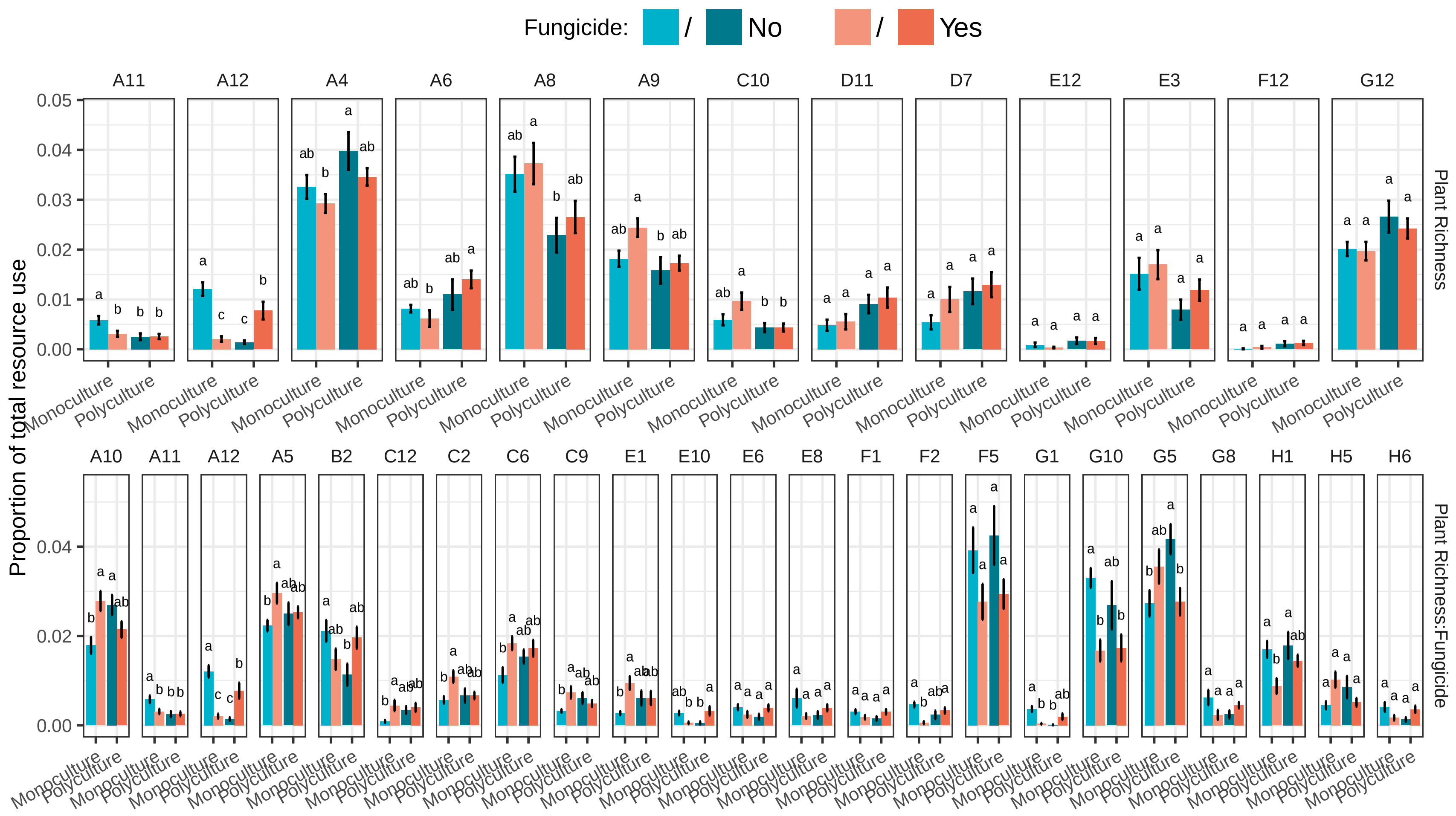

Figure S5. Proportion of total resource use for each distinct carbon source on Biolog SF-P2 plates. For each of the 95 distinct carbon sources, an ANOVA of the form `growth ~ Plant Richness / Fungicide` ws run. Only resources with significant treatment effects are shown (primary effect of plant richness (top) or nested effect of fungicide within richness (bottom)). Carbon sources are abbreviated by Biolog well location and correspond to: A10 - N-acetyl-D-glucosamine. A11 - N-actyl-b-D-mannosamine, A12 - Amygdalin, A4 - Dextrin, A5 - Glycogen, A6 - Inulin, A8 - Tween40, A9 - Tween80, B2 - D-arabitol, C10 - b-methyl-D-galactoside, C12 - a-methyl-D-glucoside, C2 - lactulose, C6 - D-mannose, C9 - a-methyl-D-galactoside, E1 - D-tagatose, E10 - p-hydroxy-phenylacetic acid, F2 - d-lactic acid methyl ester, F7 - succinic acid mono-methyl ester, G1 - l-alaninamide, G10 - Putrescine, G5 - l-asparagine, H1 - Adenosine, Error bars represent standard errors (n = 20 isolates/treatment) and different letters above boxplots indicate statistically significant differences using Tukey's HSD post-hoc test ($p < 0.05$).

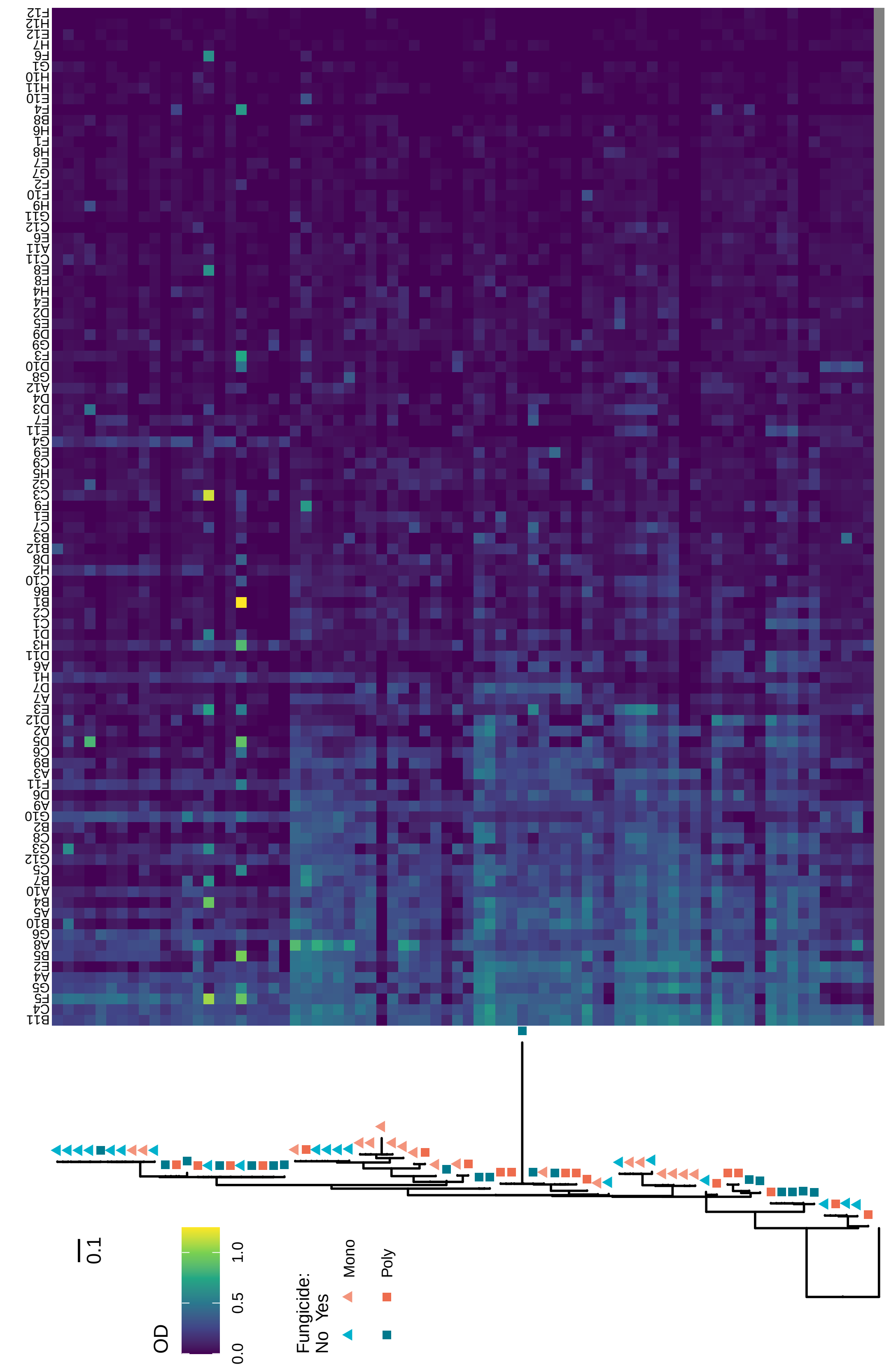

Figure S6. Resource-use profiles of each isolate, ordered according to phylogenetic relatedness. The tree here is identical to that in Figure 1, while each cell in the heatmap corresponds to that isolates measured growth (optical density) on that resource on a Biolog SF-P2 plate.

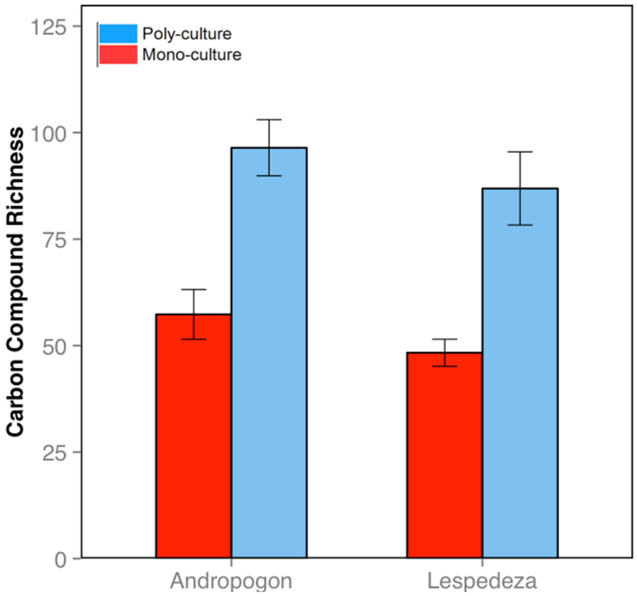

Figure S7. Effect of plant species richness on soil organic carbon (SOC) chemical richness. Soils were sampled from the rhizosphere of either Andropogon or Lespedeza plant species in monoculture (1 species) and polyculture (16 species) plots. SOC richness was determined by pyrolysis gas chromatography mass spectrometry following procedures outlined by Grandy et al. (2009). Compounds were identified using Automated Mass Spectral Deconvolution and Identification System (AMDIS V 2.65). Peaks were verified using the NIST mass spectral library and published compound libraries (Grandy et al., 2007; Grandy and Neff, 2008; Grandy et al., 2009; Stewart et al., 2011; Stewart, 2012). Richness represents the number of unique peaks identified per sample. N.b. though in the same experimental system, these results are from different samples than those reported in the main text.
